# Effects of Inhalation of Lavender Essential Oil on Open-heart Surgery Pain

**Published:** 2014

**Authors:** Armaiti Salamati, Soheyla Mashouf, Faezeh Sahbaei, Faraz Mojab

**Affiliations:** a *Faculty of Nursing & Midwifery, Islamic Azad University of Tehran Medical Branch, Tehran, Iran.*; b *Department of Pharmacognosy, School of Pharmacy, Shahid Beheshti University of Medical Sciences, Tehran, Iran. *; c *Pharmaceutical Sciences Research Center, Shahid Beheshti University of Medical Sciences, Tehran, Iran.*

**Keywords:** Aromatherapy, Lavender, Control of pain, Open-heart surgery

## Abstract

This study evaluated the effects of inhalation of lavender essential oil on the pain of open-heart surgery. The main complaint of patients after open-heart surgery is chest pain. Due to the side effects of opioids, it is important to use a non-invasive way to effectively relieve pain including aromatherapy with analgesics. This study was a clinical single-blind trial and was conducted on 40 patients who had open-heart surgery in the cardiac ICU of 2 Hospitals of Tehran University of Medical Sciences, 2012. Criteria included: full consciousness, spontaneous breathing ability and not using synthetic opioids within 2 hours before extubation. After extubation, the patients were asked to mark the intensity of their pain using the visual analogue scale. Then, a cotton swab which was impregnated with 2 drops of lavender essential oil 2% was placed in their oxygen mask, and they got breath for 10 minutes. 30 minutes after aromatherapy, they were asked to re-mark their pain intensity. The level of patient’s pain before and after aroma therapy were compared. The pain mean level before and after inhaling lavender essential oil was 5.60 (SD = 2.262) and 4.98 (SD = 2.293), respectively (p-value>0.05). Therefore, there is no significant difference and the result of study proves that lavender essential oil inhalation has no effect on reducing the pain of open-heart surgery.

## Introduction

Nowadays, heart surgery is one of the most common surgeries and has its own side effects (1). A problem in this surgery is that all heart surgeries make variable levels of pain and postoperative pain for patients which are not negligible at all (2). Researches have shown that patients that their postoperative pain is poorly controlled may experience heart failure and infection three and five times more than others, respectively (3). Moreover, open-heart surgery pain occurs during 24-72 hours after surgery (4) and it usually increases by coughing, movement, and changing position (5). Meanwhile, open-heart surgery pain causes ineffective respiration that will make a delay in leaving the bed, immobility and stagnation of blood flow. 

Developing the blood clot will increase the risk of pulmonary embolism (4). Also, acute pain effects on the immune, respiratory, cardiovascular, gastrointestinal, and endocrine systems. In addition, the postoperative pain actuates the sympathetic system that will cause to increase blood pressure, pulse, heart rate and breathing increases and becomes superficial as well. In fact, each of them increases oxygen demand required by the body, which will cause pressure on the heart muscle and subsequently, the pressure on the heart will increase. All these situations are very dangerous, especially in patients undergoing cardiac surgery (6).

Recent evidences indicated that the pain of more than 75% of open-heart surgery patients were not given appropriate and adequate care and patients had painful experiences of the time in hospital (3). Wang *et al*. also wrote that although the pain of incision was inevitable, it was controllable and in the absence of rapid and appropriate controls, it could become severe and lead to chronic pain (7). Besides, it could cause immobility, reduction of pulmonary ventilation and consequently delayed recovery, prolonged hospital admission and increasing the costs (8). Postoperative pain control is one of the major challenges for nurses. They can use pharmacological and non-pharmacological methods (9). Morphine is the most common used pain relievers after heart surgery (5) .The opioids have some unwanted side effects that may include nausea and vomiting, dizziness, drowsiness, hypotension, constipation and respiratory depression (10). It also can increase patient's tolerance to the drugs (5).

Drugs are not the only way to control pain, although they are the most effective available means for nurses (11). Due to the side effects of opioids, some procedures with low complications will be used to relieve the pain as a nursing skill (12). Conceivably, not only the use of non-invasive methods can be effective in relieving pain but also the side effects of taking too many analgesics which in many cases are threatening the health and lives of patients, can decrease. However, the patient suffering from pain can benefit from various methods of complementary medicine. Among these, aromatherapy is a technique of using volatile oils, for psychological and physical health (13). Previously, the effects of some other herb extracts were exanimate clinically (14-15).

Lavender (*Lavandula officinalis*) from labiatae family with some therapeutic properties is vastly used in the variety of aromatherapy methods (16). The primary components of lavender oil are linalool (51%) and linalyl acetate (35%). Other components include α-pinene, limonene, 1,8-cineole, *cis*- and *trans*-ocimene, 3-octanone, camphor, caryophyllene, terpinen-4-ol and avandulyl acetate (17). Applying a few drops of lavender essential oil to handkerchief and inhaling it is useful for treating insomnia, fatigue, stress and fear. Warm compress is used in menstrual cramps, stomachache, arthritis, migraine and muscle cramps (16).

 In 2006, Kim *et al*. studied on “the evaluation of aromatherapy in treating postoperative pain in New York”. They studied on the aromatherapy by lavender essential oil on 50 patients undergoing breast biopsy. The results showed postoperative lavender oil did not significantly affect pain scores. However, patients in the lavender group reported a higher satisfaction rate regarding pain control than patients in the control group (P = 0.0001) (18). In 2011, Niaz and Ali Akbar studied on “the lavender essential oil for post-cesarean pain” on 200 pregnant women. The results showed aromatherapy by lavender oil is a successful and safe complementary therapy in terms of reducing pain after cesarean (19).

It is the first assessment report of the effect of lavender essential oil on open-heart surgery pain. The researcher studied on the effectiveness of aromatherapy with Lavender essential oil on pain of open-heart surgery with fewer side effects.

## Experimental

*Methods*

This study is a single-blind clinical trial, which has the ethical approval from Ethics Committee of Islamic Azad University of Tehran Medical Branch, and was conducted on 40 patients who had undergone open-heart surgery, cardiac ICU departments in Moheb Hospital and Tehran Heart Center. The lavender oil was prepared by hydrodistillation from *Lavandula officinalis* (*L. vera*) collected from botanical garden of School of Pharmacy in Jan. 2013, and was identified (by Mr. Kamali-nejad) in Pharmacognosy Lab. in School of Pharmacy, 2% solution was prepared by sesame oil (Saman Co. Iran). Patients’ age was in the range of 18-65. Characteristic subjects of patients under investigation included: full consciousness and being aware of time and place, extubated and having spontaneous breathing, no addiction to opioids or strong analgesics, not receiving analgesics within 2 hours before intervention, not having asthma, allergies, chronic obstructive pulmonary and other lung diseases, contact dermatitis to aromatic substances. Also, some exclusion criteria were set in this research. The patients that needed medical intervention during the inhalation, or in case of allergies, respiratory problems and emergencies, were excluded from this research.

Gradual sampling was used in this study. A two-part questionnaire was used in this study for the data collection. The first part included demographic characteristics subjects under investigation (age, gender, income, education, marital status, place of residence, *etc*.) and the second part was a visual analogue scale to determine the pain intensity on a 10 cm ruler including 10 numbers begin form 0 indicates "no pain" and ends in 10 that indicates "most severe pain".

**Figure 1:**
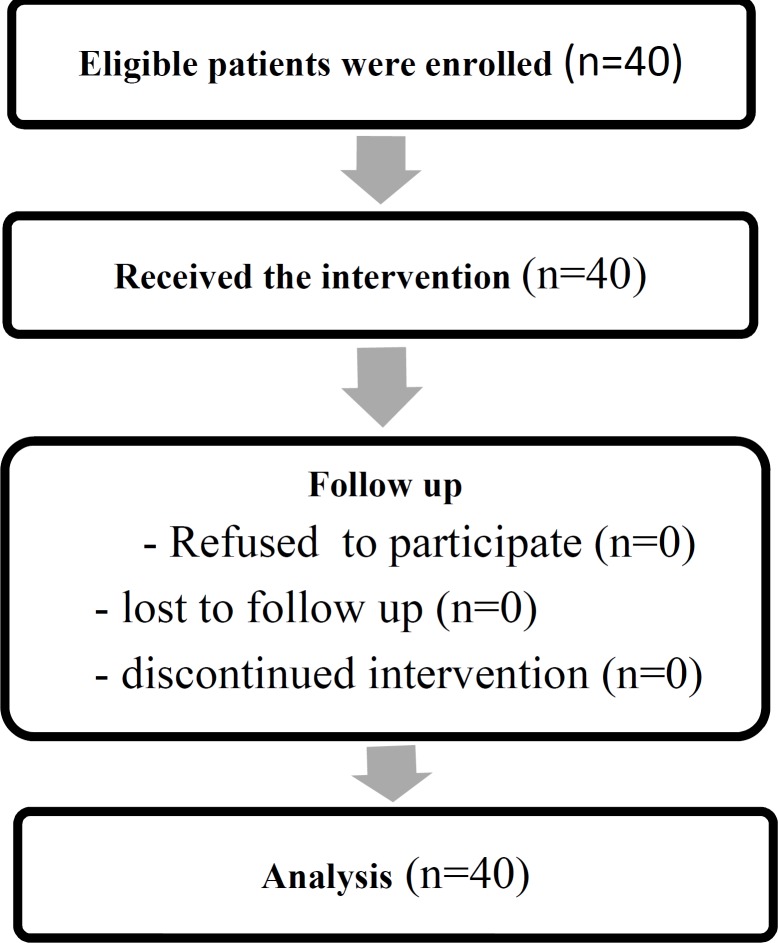
Follow-up of patients

 All patients who consented to the study received a lavender oil patch test, preoperatively. One drop of lavender oil was dropped to their ankle. In order to reduce inhalation and skin absorption of the lavender, the test patch was immediately covered with an occlusive dressing and removed after a two-minute exposure. Then, the researcher was present in the cardiac ICU in the day of surgery. After extubation, the patients were asked to intensity of their pain using the visual analogue scale. Then a cotton swab which was impregnated with 2 drops of lavender 2% was placed in their face oxygen mask, and they breathed that for 10 minutes. 30 minutes after, the researcher asked their pain intensity (Figure 1). SPSS 18.0 (SPSS Inc, Il, USA) was used for statistical analysis. The level of patient’s pain before and after aromatherapy were analyzed by paired t-tests and Chi-square and Fishers’ exact is used to investigate the relationship between demographic characteristics and pain. Statistical significance was accepted for P < 0.05. 

## Results

Demographic data from participants showed that 57.5% of samples were males, majority of samples (47.5%) were 50 to 59 years and average age was 50 years old (SD=15.16). 37.5% of patients had hypertension, 30% had hyperlipidemia disease and 20% had diabetes. 57.5% had an experience of previous surgery and only 10 % had an experience of aromatherapy. None of the subjects had experience of using lavender essential oil, previously.

Comparison of pain average before and after aromatherapy showed that the pain mean level before inhaling lavender essential oil was 5.60 (SD= 2.262), and after inhaling lavender was 4.98 (SD=2.293). Since the p-value=0.1>0.05, there is no statistically significant difference and the result of study proves that lavender essential oil inhalation has no effect on reducing the pain of open-heart surgery (Table 1).

**Table 1:** Pain average comparison before and after aromatherapy

Aromatherapy	Average	SD
Before	5.60	2.262
After	4.98	2.293

Among the demographic variables using Chi-square statistical test, only age and marital status had a significant effect on pain. The elder people and married people felt less pain. There were no lavender essential oil related adverse effects hemodynamic, respiratory, gastrointestinal or allergic reaction observed during the study.

## Discussion

One of the factors affecting the pain is age; Potter and Perry have studied on “relation of developmental differences and how adults reaction to pain are more effective” (20). In our study, 47.5% were 50 to 59 years, average age was 50 years old (SD=15.16) that using the Chi-square test revealed the effect of age on pain. Elder people felt less pain. Some researchers believe the pain threshold and tolerance levels are higher in men (11). Therefore, in this study gender were considered, but the difference was not significant (p=0.8). Also, using statistical testing showed that married peoples felt less pain (p=0.008). Patients with previous surgery can affect the response to pain (6) but results in this study the results showed no statistically meaningful difference (p=0.67). Other demographic characteristics did not show any meaningful differences. Finally, the pain mean level showed no meaningful difference before and after aromatherapy. So, the Lavender inhalation did not effect on pain after open-heart surgery.

In 2006, Kim *et al.* studied on the "Evaluation of aromatherapy in treating postoperative pain in New York” (18). Similar research has been done in this case that the results are in accordance with results of that study. The results of this study support previous work that indicated aromatherapy does not cause a detectable analgesic effect (21), But in 2007, Kim *et al.* has done another study with the title "Treatment with lavender aromatherapy in the post-anesthesia care unit reduces opioid requirements of morbidly obese patients undergoing laparoscopic adjustable gastric banding" that showed significantly more patients in the placebo group required analgesics for postoperative pain (22.27, 82%) than patients in the lavender group (12.26, 46%) (P=0.007). Moreover, the Lavender group patients required significantly less morphine postoperatively than placebo group patients (22). Also, in 2011, Niaz and Ali Akbar studied on the "lavender essence for post-cesarean pain". They concluded aromatherapy by using Lavender oil and it was a successful and safe complementary therapy in reducing pain after cesarean (19).

Essential oils have both physiological and psychological effects (23). When lavender oil is inhaled for 10 minutes, there is an increase in blood flow rate and a decrease in galvanic skin conduction and systolic blood pressure (indicating a reduction in sympathetic nerve activity) (24). Human studies with lavender have demonstrated a significant relaxation effect and reduced anxiety, but no direct antinociception (25, 26).
